# LINKING LIVES: DISRUPTING THE CYCLE OF SOCIAL ISOLATION

**DOI:** 10.1093/geroni/igac059.1421

**Published:** 2022-12-20

**Authors:** Karen Fredriksen Goldsen, Hyun-Jun Kim, Charles Hoy-Ellis, Christi Nelson

**Affiliations:** University of Washington, Seattle, Washington, United States; University of Washington, Seattle, Washington, United States; University of Utah, Salt Lake City, Utah, United States; University of Washington, Seattle, Washington, United States

## Abstract

Loneliness has been found to be associated with increased risk for early mortality and dementia, with sexual and gender diverse older adults at elevated risk of both social isolation and loneliness. Based on the Health Equity Promotion Model and Iridescent Life Course, we examine factors associated with increased risk of loneliness over time, utilizing 2014 to 2016 data from the Aging with Pride: National Health, Aging, Sexuality/Gender Study, a longitudinal national study of LGBTQ+ midlife and older adults. The findings illustrate that sexual and gender diverse older adults had nearly double rates of loneliness compared to the general population, with those living alone and having cognitive decline at increased risk. We found that higher mastery, LGBTQ+ community engagement, larger network size, and being partnered/married were associated with less loneliness over time. Loneliness is ripe for the development of interventions; additional longitudinal data is needed to further assess trajectories in loneliness.

